# Kinetics of volume expansion during a fluid challenge

**DOI:** 10.1186/cc13343

**Published:** 2014-03-17

**Authors:** H Aya, A Rhodes, M Grounds, M Cecconi

**Affiliations:** 1 St George's Healthcare NHS Trust, London, UK

## Introduction

According to Guyton and colleagues [1], expansion of blood volume can increase cardiac output (CO) in so far as the change in volume affects the mean systemic filling pressure (Pmsf). However, rapid stress relaxation occurs after acute intravascular volume expansion so that the effect of volume on Pmsf and CO may disappear after a few minutes. The objective of the present study is to describe the extent of the haemodynamic changes generated by a fluid challenge during 10 minutes on critically ill patients.

## Methods

Patients admitted to the ICU were monitored with a calibrated LiDCOplus (LiDCO, UK) and Navigator (Applied Physiology, Australia) to estimate Pmsf analogue (Pmsa). Then 250 ml Hartmann's solution or Volplex was infused over 5 minutes. Data were recorded automatically from baseline to 10 minutes after the end of fluid infusion. The time to maximum response and percentage change between baseline and last value of different haemodynamic parameters are also reported.

## Results

**Figure 1:**
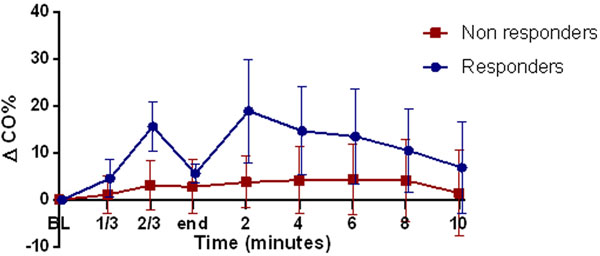
**Change of CO during and after a fluid challenge**. BL, baseline; end, end of fluid infusion.

Sixty fluid challenges were studied in 34 patients, with a mean duration of 5.3 minutes (± 2.5). In 22 events (37%), CO increased more than 10% (responders). The change between baseline and last value was greater in nonresponders for heart efficiency (Eh) (-9.2% ± 9.7 vs. -3.1% ± 13.9, *P *= 0.05) but not in other haemodynamic variables. Time to maximal response on CO was 2 minutes after the end of the infusion (Figure 1).

## Conclusion

Stress relaxation damps down the effect of a fluid challenge after 10 minutes except in terms of heart efficiency. The effect of a fluid challenge should be assessed up to 2 minutes after the end of fluid infusion. Failure to do so may mislead clinicians about the patient's fluid responsiveness.
